# *Mortierella wolfii*–Associated Invasive Disease

**DOI:** 10.3201/eid2009.140469

**Published:** 2014-09

**Authors:** Nathalie Layios, Jean-Luc Canivet, Frédéric Baron, Michel Moutschen, Marie-Pierre Hayette

**Affiliations:** University Hospital of Liege, Liege, Belgium

**Keywords:** Mortierella, molecular diagnosis, mucormycosis, chronic granulomatous disease, fungi, mold, primary immunodeficiency

**To the Editor:** In January 2013, a 34-year-old man was admitted because of severe shock to the intensive care unit (ICU) at University Hospital of Liege (Liege, Belgium) on day 10 after nonfamilial hematopoietic stem cell transplantation (HSCT). X-linked *gp91phox* gene mutation chronic granulomatous disease (CGD) had been diagnosed when he was 4 years of age. The patient had a history of recurrent pulmonary aspergillosis (*Aspergillus fumigatus* infection) and anaphylaxis to lipid-based formulations of amphotericin B (AmB). During the year before HSCT, he had received voriconazole for possible recurrent aspergillosis until 2 cavitary necrotic pulmonary lesions prompted prolonged combined treatment with caspofungin. No fungus was yielded before transplant. 

On day 9 after HSCT, a computed tomographic (CT) scan of the abdomen showed multiple hypodense lesions in the hepato-splenic parenchyma, and the patient was admitted to the ICU. Antifungal drug administration were escalated (voriconazole 6 mg/kg twice daily, caspofungin 70 mg/day), taking into account patient’s history of anaphylaxis. The patient recovered from aplasia within 12 days (*1*), and CT-guided liver biopsy was performed on day 20 after HSCT.

Microbiological cultures of the liver biopsy sample were performed on 5% sheep blood agar and Sabouraud agar medium supplemented with chloramphenicol. The plates were incubated at 30°C and 37°C, respectively, for 10 days. Subcultures were performed on malt agar, Takashio, and agar-agar medium and incubated at 30°C, 37°C, and 42°C, respectively, to obtain sporulation. Blood cultures were collected on the same day the biopsy was performed on BacT/ALERT FAN aerobic and anaerobic medium bottles (bioMérieux, Durham, NC, USA). Antifungal susceptibility testing was performed by Etest. Histopathologic examination was conducted after the liver sections were stained with hematoxylin and eosin, periodic acid–Schiff, and Gomori methenamine silver.

We sequenced regions of internal transcribed spacers (ITS) 1 and 2 of the rRNA genes (*2*).The ribosomal target of the large subunit RNA gene (D1–D2 region) was used to confirm the first results. Sequences were aligned by using the GeneStudio Pro software (http://genestudio.com/) and identified in BLAST (http://blast.ncbi.nlm.nih.gov/Blast.cgi) and in the CBS database for filamentous fungi (http://www.cbs.knaw.nl/collections/BioloMICSSequences.aspx).

Histologic examination of the liver biopsy sample showed fungal filaments of variable diameter, without septa, and with bulbous dilations suggesting mucormycosis amid necrotic parenchyma (Figure). A white filamentous mold grew after the sample was incubated for 2 days on Sabouraud agar at 30°C and 5% sheep blood agar medium at 37°C. Microscopic examination of the culture confirmed a fungus belonging to the mucoromycotina. Nonseptate hyphae of irregular width were visible and marked by short lateral extensions distributed at right angles along the filaments. Despite subcultures on different media and incubation at 30°C, 37°C, and 42°C, no sporulation was observed. The fungus did not grow on Sabouraud agar supplemented with cycloheximide. One blood culture was positive after 3 days’ incubation at 37°C, and the subculture grew the same fungus. In vitro susceptibility testing showed high MICs (>32 mg/L) for voriconazole and caspofungin and lower MICs for AmB (1 mg/L) and posaconazole (0.012 mg/L). The 3 targeted sequences of ITS2 alone (GenBank accession no. KJ825897), ITS1–ITS2 (GenBank accession no. KJ82598), and D1–D2 region of the rRNA genes (GenBank accession no. KJ825899) identified *Mortierella wolfii* with 98% and 100% similarity with the reference sequences in the CBS database.

**Figure:**
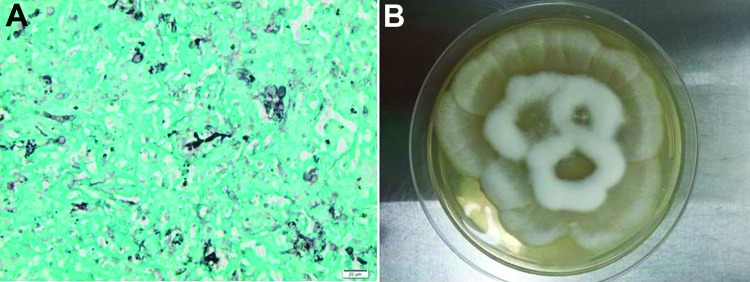
Histopathologic findings from a 34-year-old man with X-linked *gp91phox* gene mutation chronic granulomatous disease. A) Liver biopsy results showing numerous irregular and nonseptate hyphae with bulbous dilations. Gomori methenamine silver stain. Original magnification ×400. B) Culture of *Mortierella wolfii*, after 3-days of incubation at 30°C on Sabouraud agar medium.

CGD is a primary immunodeficiency syndrome characterized by impaired phagocytic activity of intracellular pathogens and fungi. Invasive fungal infections account for one third of deaths attributed to CGD, and lung involvement is predominant (*3*). To our knowledge, the 2 reports of human cutaneous infection with *M. wolfii* are considered inconclusive (*4*). It is a mold belonging to the order Mortierellales according to the most recent taxonomy (*5*) and is considered a pathogen solely of animals. It causes abortion, encephalitis, and pneumonia in cattle in specific geographic locations (Australia, North America, and Japan) (*6*). The natural habitats of *M. wolfii* include moldy grass in silage, and infected animals might inhale spores from contaminated silage or acquire them through digestive tract ulcerations after ingestion of semen (*4*). Thus, possible transmission routes of *Mortierella* sp. in this patient include airborne exposure to mulch or ingestion of contaminated imported food during pressure-selection azole prophylaxis and inflammatory bowel disease. After being ingested or inhaled, this weakly virulent mold must have remained quiescent until a few months before HSCT. We suspect that it was responsible for the necrotic cavitary pneumonia for which no fungus was identified before transplant. *M. wolfii* eventually emerged during a profound iatrogenic neutropenic period (*1*). Because death partly correlates with dissemination, preemptive and adequate antifungal treatment is of utmost importance in mucormycosis. In this patient, who died 11 days after ICU admission, past anaphylaxis precluded prompt initiation of a lipid-based formulation of AmB, which remains the best choice for treating invasive mucormycosis (*7*). Posaconazole, a second-choice drug, has shown efficacy in CGD patients who had invasive mucormycosis resistant to first-line treatment (*8*). Allogeneic donor–matched HSCT has a curative potential in CGD patients with refractory fungal infections (*9*). Several other authors have pointed to the emergence of rare new fungi in CGD, as well as reclassification of misdiagnosed fungi, identified by sequence-based analysis (*10*).
